# Silencing PROK2 Inhibits Invasion of Human Cervical Cancer Cells by Targeting MMP15 Expression

**DOI:** 10.3390/ijms21176391

**Published:** 2020-09-02

**Authors:** Min-Hua Wu, Pei-Ru Wu, Yi-Hsien Hsieh, Chia-Liang Lin, Chung-Jung Liu, Tsung-Ho Ying

**Affiliations:** 1 Laboratory Department, Chung-Kang Branch, Cheng-Ching General Hospital, Taichung 40764, Taiwan; 3150@ccgh.com.tw; 2 Department of Medicinal Botanicals and Health Applications, Da-Yeh University, Chunghua 51591, Taiwan; 3 Department of Pathology, Cheng-Ching General Hospital, Taichung 40764, Taiwan; peiruwu5@gmail.com; 4 Institute of Medicine, Chung Shan Medical University, Taichung 40201, Taiwan; hyhsien@csmu.edu.tw (Y.-H.H.); hiking003@hotmail.com (C.-L.L.); 5 Division of Gastroenterology, Department of Internal Medicine, Kaohsiung Medical University Hospital, Kaohsiung 80708, Taiwan; 6 Regenerative Medicine and Cell Therapy Research Center, Kaohsiung Medical University, Kaohsiung 80708, Taiwan; 7 Department of Obstetrics and Gynecology, Chung Shan Medical University Hospital, Taichung 40201, Taiwan; 8 Department of Obstetrics and Gynecology, School of Medicine, College of Medicine, Chung Shan Medical University, Taichung 40201, Taiwan

**Keywords:** PROK2, cervical cancer, migration, invasion, MMP15

## Abstract

Cervical cancer is the second most frequent type of gynecologic cancer worldwide. Prokineticin 2 (PROK2) is reported to be involved in tumor progression in some malignant tumors. However, the role of PROK2 in the development of cervical cancer remains unknown. Our results indicate that PROK2 is overexpressed in the human cervical cancer. Cervical cancer patients with high PROK2 expression have a shorter overall survival rate (OS) and disease-free survival rate (DFS). PROK2 acts as a potential biomarker for predicting OS and DFS of cervical cancer patients. We further show that PROK2 is important factor for oncogenic migration and invasion in human cervical cancer cells. Knockdown PROK2 significantly inhibited cell migration, invasion, and MMP15 protein expression in HeLa cells. High expression of MMP15 is confirmed in the human cervical cancer, is significantly associated with the shorter overall survival rate (OS) and is correlated with PROK2 expression. Overexpression of PROK2 using PROK2 plasmid significantly reverses the function of knockdown PROK2, and further upregulates MMP15 expression, migration and invasion of human cervical cancer cells. In conclusion, our findings are the first to demonstrate the role of PROK2 as a novel and potential biomarker for clinical use, and reveal the oncogenic functions of PROK2 as therapeutic target for cervical cancer.

## 1. Introduction

Cervical cancer is one of the most common cancers in worldwide women, including among Taiwanese women. There is abundant evidence that human papillomaviruses (HPV) are well-known as a major risk factor for cervical cancer and may increase the possibility of pre-cancerous cervical lesions [1]. Recurrence and progression of cervical cancer mainly leads to death, despite treatment including surgery, chemotherapy and radiation therapy or even combinations of these strategies [2]. High resistance to chemotherapeutic reagents and adaptability to radiotherapy contribute to cervical cancer-induced serious lethality [3]. Therefore, it is urgent need to identify potential biomarkers for prognosis and novel therapeutic targets for cervical cancer. 

Metastasis is still a critical issue for cancer prevention and target therapy. Metastasis is the major cause leading to the poor clinical outcomes and the high mortality in cervical cancer. Metastasis exacerbates tumor progression through a complex process that involves the destruction of the extracellular matrix (ECM), the increase in tumor cell migration and invasion from the primary tumor site to target organ. ECM degradation by extracellular proteinases contribute to tumor cell invasion, metastasis and malignant progression [4]. Among the proteolytic proteinase systems, matrix metalloproteinases (MMPs) act as the critical factors responsible for ECM degradation [4,5]. MMP15 is high expressed in the primary and metastatic melanoma cells [6]. MMP15 contributes to HBXIP-facilitated metastasis of hepatocellular carcinoma cells [7]. MMP15 regulates LncRNA MAFG-AS1-promoted the aggressiveness of breast carcinoma [8]. 

Prokineticin 2 (PROK2), a member of prokineticin protein family, is a cysteine-rich secreted protein containing a conserved N-terminal sequence of AVITGA and 10 cysteines [9]. Studies have shown that PROK2 might be involved in tumorigenic processes through regulating angiogenesis in glioblastomas [10] and in colorectal cancer [11], myeloid cell infiltration in pancreatic cancer [12], growth in hepatocellular carcinoma [13] and tumor progression in prostate cancer [14]. PROK2 might be related to drug resistance of breast cancer and metastasis to lung [15]. PROK2 expression was considered as a potential prognostic biomarker for human colorectal cancer [16]. Based on these evidences, PROK2 is involved in the tumorigenesis and target therapy. However, the biological function, underlying molecular mechanism and clinical significance of PROK2 in human cervical cancer has not been explored.

## 2. Results

### 2.1. PROK2 Is Overexpressed in Cervical Cancer and Is Associated with Poor Survival

**Figure 1:**
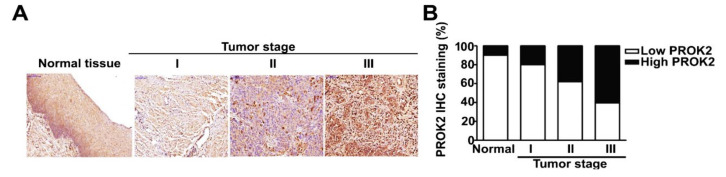

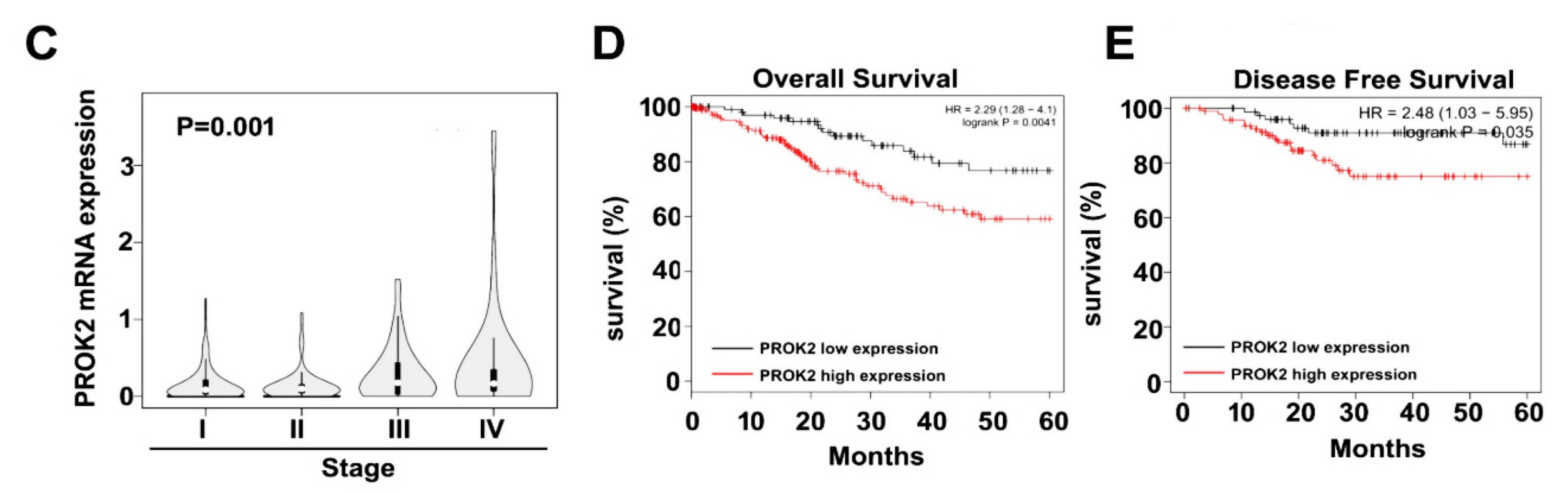
The expression of PROK2 in cervical cancer tissues and Kaplan–Meier analysis of cervical cancer patients’ survival rates in association with PROK2 expression. (**A**,**B**) Representative IHC staining of PROK2 in matched cervical cancer tissues and adjacent noncancerous cervical tissues with different staining intensity. (**C**) Validation of PROK2 expression based on the GEPIA databases for representative examples. (**D**) Overall survival rate (OS) and (**E**) Disease free survival rate (DFS) in patients with high or low PROK2 expression. The red line indicates high expression, and black line indicates low expression.

To investigate the potential role of PROK2 in cervical cancer, we analyzed PROK2 expression in human cervical cancer tissues. We performed IHC staining to measure PROK2 protein expression in paraffin-embedded tissue samples of human cervical tumors. PROK2 was highly expressed in cervical cancer tissue, particularly in patients at stage III. PROK2 expression was positively correlated with tumor-node-metastasis (TNM) stage. In contrast, PROK2 was slightly expressed in the normal tissues (Figure 1A,B). GEPIA was used to further confirm the high expression of PROK2 in patients with the advanced stages (III and IV) of human cervical cancer (Figure 1C). A log-rank test and Kaplan-Meier analysis were used to identify the effect of PROK2 on the survival of patients with cervical cancer. The results indicated that patients with high PROK2 expression have significantly shorter overall survival (OS) (HR = 2.29, 95% CI 1.28–4.1, *p* = 0.0041) and disease-free survival (DFS) (HR = 2.48, 95% CI 1.03–5.95, *p* = 0.035) than those with low PROK2 expression (Figure 1D,E).

### 2.2. Effect of PROK2 on Cell Viability and Cell Cycle Regulation in Human Cervical Cancer Cells

**Figure 2:**
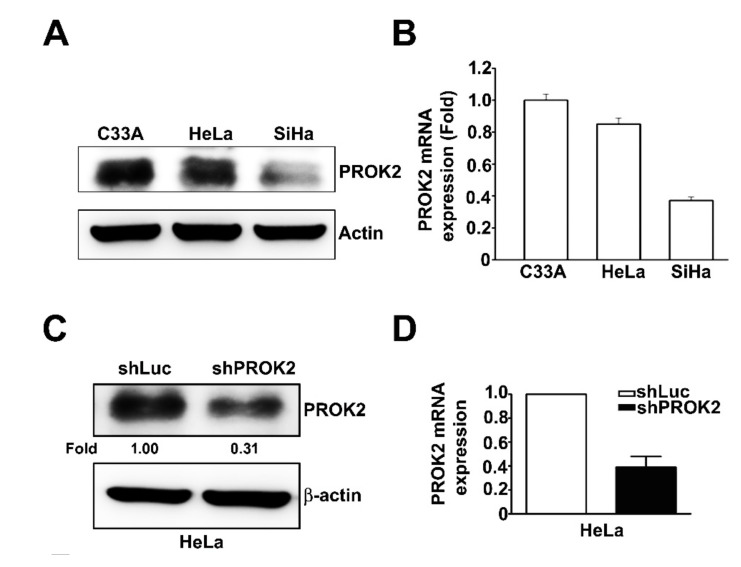

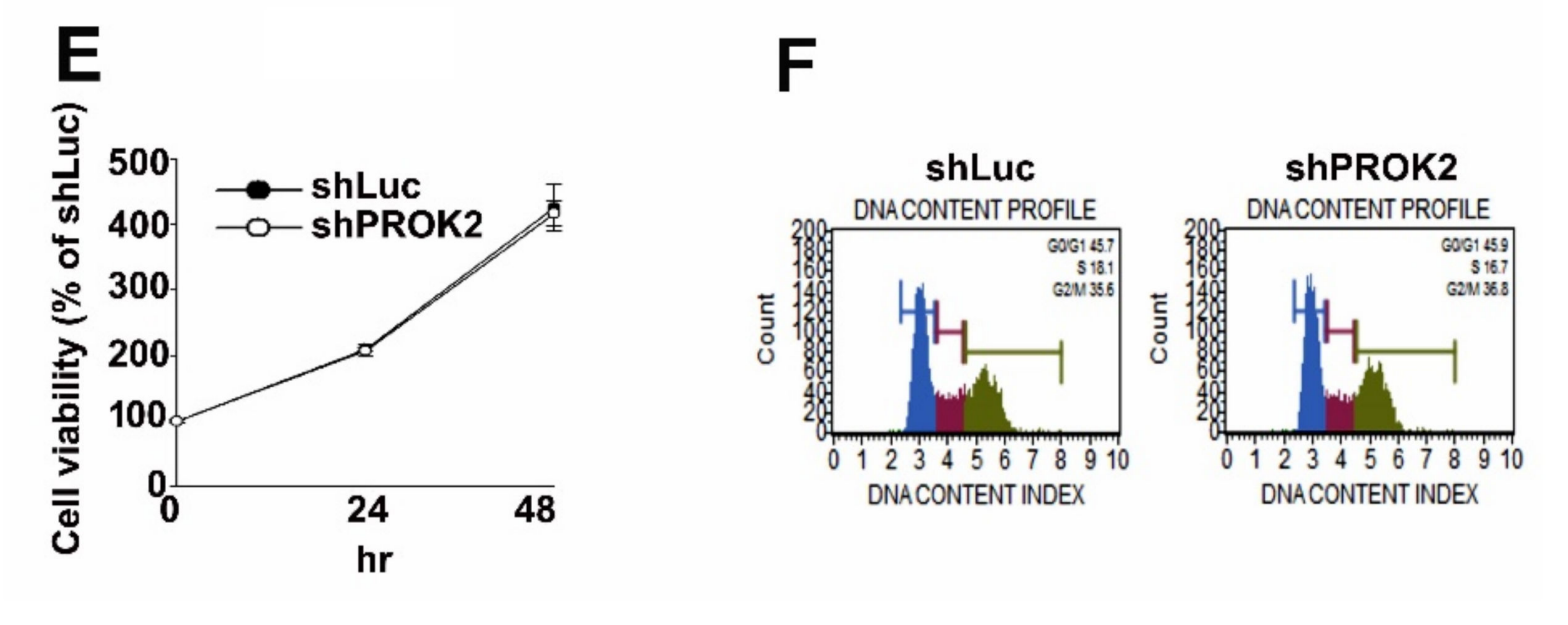
Effect of knockdown PROK2 on cell viability and cell cycle in human cervical cancer HeLa cells. (**A**,**B**) Immunoblotting and RT-qPCR analysis of PROK2 protein and mRNA expression in three cervical cancer cell lines (C33A, HeLa and SiHa). β-actin as a protein loading control, GAPDH as a mRNA loading control. (**C,D**) The protein and mRNA expression of PROK2 in shLuc- or shPROK2-HeLa cells. (**E**) Cell viability of shLuc- or shPROK2-HeLa cells was measured by cell viability assay at 24 h and 48 h after seeding. (**F**) Cell cycle distribution of shLuc- or shPROK2-HeLa cells were measured by flow cytometry.

To examine the PROK2 expression in three cervical cancer cells lines (C33A, HeLa and SiHa). As shown in Figure 2A and Figure 2B, we found that higher mRNA and protein expression of PROK2 in C33A and HeLa cells than in SiHa cells. To investigate the effects of PROK2 on cell proliferation and cell cycle on HeLa cells, we infected cervical cells with PROK2 shRNA to generate PROK2 shRNA-stable cervical cancer cells. The knockdown efficiency was confirmed by western blotting and RT-qPCR revealing that the protein and mRNA expressions of PROK2 were significantly reduced in shPROK2-HeLa cells, compared with that of shLuc-HeLa cells (Figure 2C,D). Cell viability and cell cycle were further measured in these shLuc- or shPROK2-HeLa cells. We observed that knockdown PROK2 has no effects in regulating cell viability and cell cycle arrest induction through cell viability assay and PI staining by flow cytometry analysis (Figure 2E,F) in both shLuc- or shPROK2-HeLa cells. These results suggest that cell viability of human cervical cancer HeLa cells not regulated by PROK2.

### 2.3. Knockdown of PROK2 Inhibits the Cell Migration and Invasion

**Figure 3:**
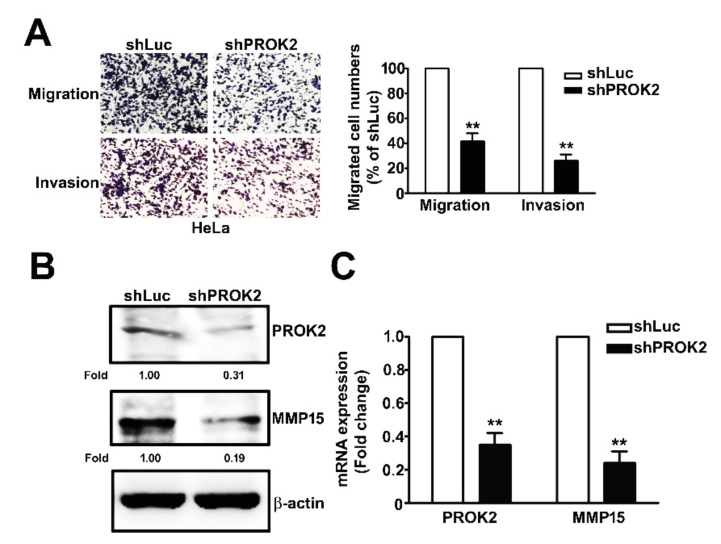

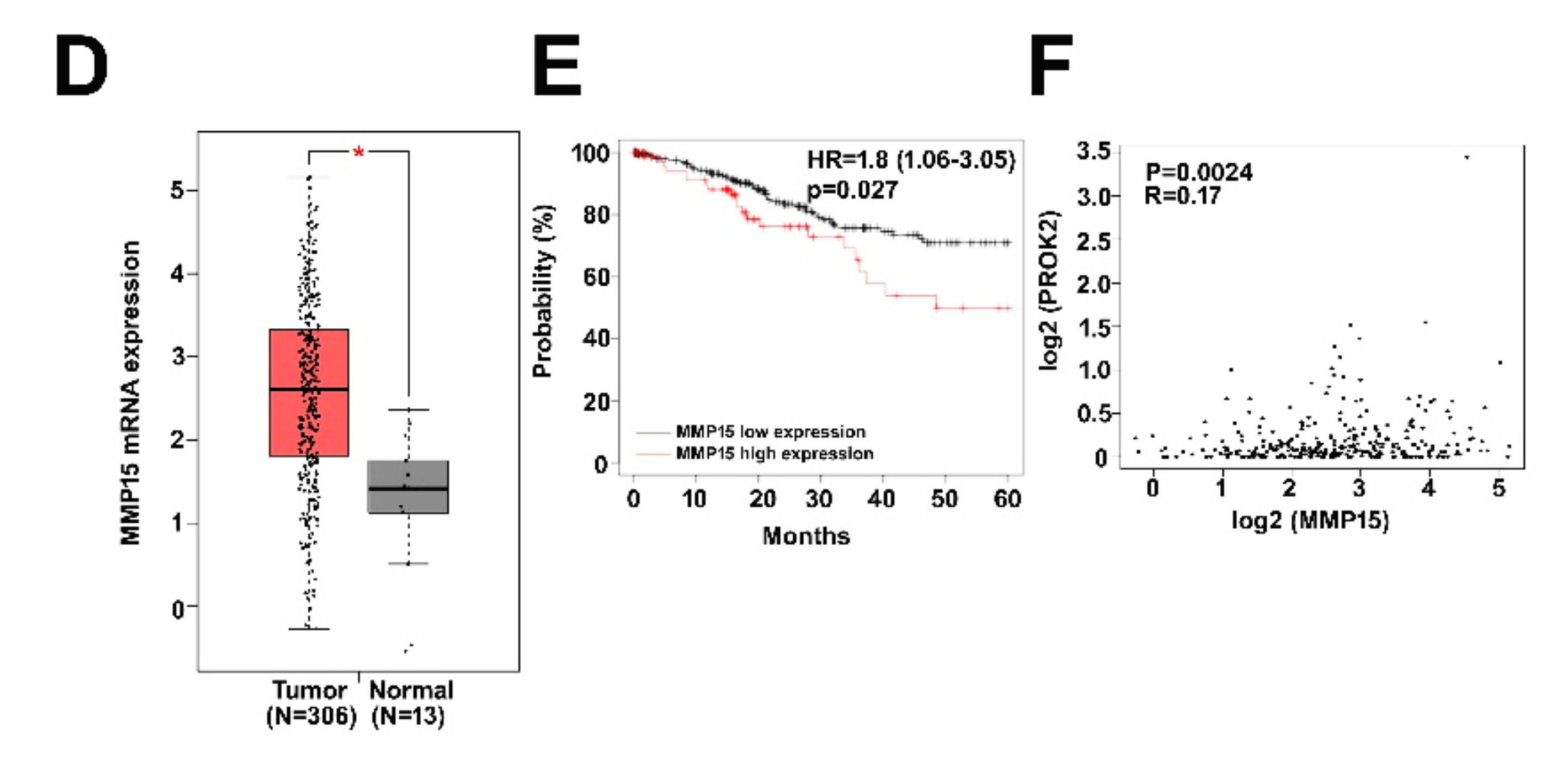
Effect of knockdown PROK2 on MMP15 expression and cell invasion in human cervical cancer HeLa cells. (**A**) Human HeLa cells were transfected with or without PROK2 shRNA, then followed by measuring the capacity of cell migration and invasion. (**B**,**C**) The protein and mRNA expression of MMP15 were inhibited by shPROK2-HeLa cells were measured by western blotting and R-qPCR assay. (**D**) Validation of MMP15 gene expression in matched cervical cancer tissues and adjacent noncancerous cervical tissues from the GEPIA databases. T: cervical tumour tissue (*n* = 306); N: normal cervical tissue (*n* = 13), * *p* < 0.05 versus normal cervical tissue. (**E**) Overall survival rate (OS) in patients with high or low MMP15 expression. The red line indicates high expression, and black line indicates low expression. (**F**) MMP15 expression was correlated with PROK2 expression in human cervical cancer patients. ** *p* < 0.01 versus shLuc cells.

Our studies indicated that PROK2 is high expressed in the advanced stages (III and IV) of cervical cancer, and is positively correlated with poor survival of patients. We further used the in vitro migration and invasion assay to identify the role of PROK2 in regulating cell migration and invasion of human cervical cancer cells. PROK2 knockdown by shRNA attenuated the capacity of migration and invasion in human HeLa cervical cancer cells (Figure 3A).

### 2.4. Knockdown of PROK2 Reduced the Expression of MMP15 and is Associated with Poor Survival of Human Cervical Cancer Patients

MMP15 has been shown to play an important role contributing to cancer progression through upregulating the capacity of migration and invasion in cancer cells [17]. We measured the effect of PROK2 on protein and mRNA expressions of MMP15 in human cervical cancer HeLa cells by using RT-qPCR and immunoblot assays. The results showed that the protein and mRNA levels of MMP15 were significantly reduced PROK2 shRNA (Figure 3B,C). GEPIA database was also used to further confirm the high mRNA level of MMP15 in human cervical cancer tissues compared with those in the normal cervical tissues (Figure 3D). In addition, we also revealed that cervical cancer patients with high expression of MMP15 had a shorter overall survival (OS) (HR = 1.8, 95% CI 1.06–3.05, *p* = 0.027) than those with low MMP15 expression (Figure 3E). The relation between MMP15 and PROK2 expressions have been further investigated in human cervical cancer. MMP15 high expression is positively correlated with PROK2 expression (R = 0.17, *p* = 0.0024; Figure 3F). This observation suggests that MMP15 expression involved in PROK2 regulated cell migration and invasion of human cervical cancer cells.

### 2.5. Knockdown PROK2 Inhibits Cell Migration and Invasion of Human Cervical Cancer HeLa Cells Through Targeting MMP15

**Figure 4:**
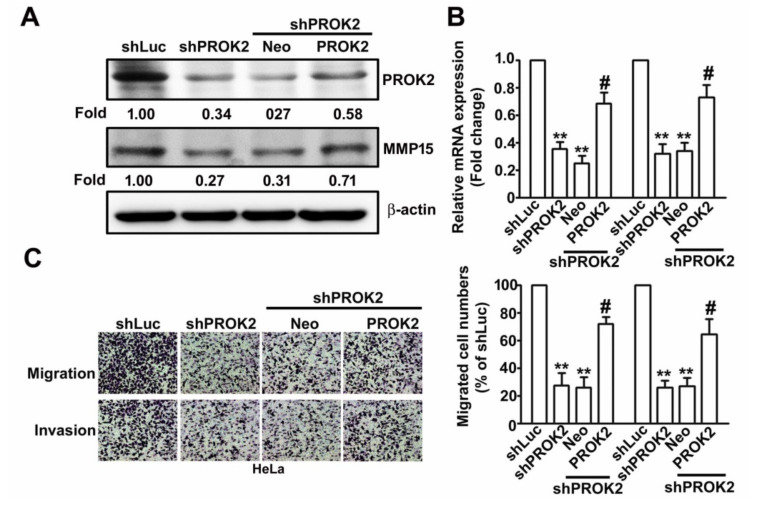
MMP15 involved in PROK2 regulates cell migration and invasion in human cervical cancer HeLa cells. Using transfected with Neo or PROK2 overexpression plasmid in shLuc- or shPROK2-HeLa cells for 48 h. (**A**) The protein expression of MMP15 and PROK2 were measured by the western blotting. β-actin as a protein loading control. (**B**) The MMP15 and PROK2 mRNA expression were detected by RT-qPCR assay. GAPDH as a mRNA loading control. (**C**) In vitro migration and invasion assay was conducted to measures the cell migration and invasion numbers. ** *p* < 0.01 versus shLuc cells; # *p* < 0.05 verus shPROK2 cells.

To evaluate the effect of PROK2 on human cervical cancer HeLa cells thorough regulation of MMP15 expression. We co-transfected without or with PROK2-overexpressing vector in shLuc- or sh-PROK2-HeLa cells were detected with western blotting and RT-qPCR assay. We found that upregulates the protein and mRNA expression of MMP15 mediated in shPROK2-HeLa cells were rescued by PROK2 overexpression (Figure 4A,B). The inhibitory effects of knockdown PROK2 on cell migration and invasion were restored by PROK2 overexpression in shPROK2-HeLa cells, compared with Neo-transfected cells (Figure 4C). In conclusion, we demonstrated that PROK2 regulation of cell migration and invasion of human cervical cancer cells by targeting MMP15.

## 3. Discussion

Cervical cancer remains as one of most common cancers in women. Cervical cancer still has a low survival rate and is a major cause of cancer-related deaths around the world. So far, optimal treatments for cervical cancer including chemotherapy, surgical resection, and radiotherapy still remain clinically unmet needs. Therefore, it is necessary to identify potential predictors for prognosis, and novel therapeutic targets for cervical cancer. In the present study, we observed the following results: (i) PROK2 is high expressed in cervical cancer and is positively correlated with shorter OS and DFS of cervical cancer patients; (ii) PROK2 knockdown by shPROK2 significantly decreases the capacity of migration and invasion in human cervical cancer cells; (iii) PROK2 knockdown shows no influence on cell growth, the induction of cell cycle arrest or apoptosis; (iv) MMP15 expression is positively regulated by PROK2. Cervical cancer patients with MMP15 overexpression have a shorter OS; (v) PROK2-expressing vector reverses PROK2 shRNA-inhibited MMP15 expression, migration and invasion of cervical cancer cells.

These results demonstrate that PROK2 might be a novel prognostic factor for OS and DFS of cervical cancer patients. Change of MMP15 expression by PROK2 may contribute to cervical cancer metastasis. PROK2 has been reported to act as an important factor in the creation of tumor microenvironment favoring tumor development and progression. In the cohort studies, high PROK2 expression is found in human colorectal cancer (CRC) tissues and is positively correlated with lymphatic invasion, lymph node metastasis, clinical stage, and postoperative liver recurrence rate. Recurrence-free survival (RFS) duration is significantly shorter in patients with PROK2-positive CRC than in those with PROK2-negative CRC [16]. PROK2 and PROKR receptors are observed to be expressed in malignant prostate epithelial cancer [14]. PROKR2 transcripts are significantly high expressed to activate prokineticin signaling and inhibit apoptosis in the advanced stages of neuroblastoma, which contributes to human neuroblastoma progression [18]. In this study, we first reported that PROK2 is high expressed in cervical cancer, particular in the advanced stages (III and IV). Cervical cancer patients with high PROK2 expression have a significant shorter OS rate and DFS rate. PROK2 could be considered as a potential prognostic biomarker and therapy target for human cervical cancer.

Remodeling of ECM and subsequent cellular migration and invasion contribute to the distant metastasis of cancer cells and malignant tumor progression. PROK2 is show to promote liver metastasis of human colorectal cancer cells in the nude mice tumor xenograft model [16]. Blocking PROK2 with antibodies results in a significant reduction in pancreatic tumor regrowth, angiogenesis, and metastasis in weekly gemcitabine-treated mice. Both PROK2 inhibition and metronomic chemotherapy might be used as legitimate ‘add-on’ treatments for preventing post-chemotherapy pancreatic cancer recurrence, progression, and metastasis following weekly gemcitabine therapy in the future [12]. PROK2-expressing neutrophils are involved in the 5-fluorouracil-induced aggravation of breast cancer metastasis to lung [15]. PROK2 could be involved in prostate carcinogenesis and could be useful for prostate cancer outcome evaluation, and as a target for prostate cancer treatment [14]. Use of PROK2 antagonist suppresses the tumorigenic processes through inhibiting angiogenic process in glioma and blocking myeloid cell infiltration in pancreatic cancer [10]. Colorectal cancer cells with PROK2 siRNA show a significant decrease in PROK2 expression and tumor growth in mice models [11]. MicroRNA-374a acts as tumor suppressor to inhibit cell proliferation, cell cycle progression, and cell invasion through targeting PROK2 in human glioma cells [19]. In the present study, we observed that PROK2 is required for the migration and invasion of human cervical cancer cells. The capacity of migration and invasion in human HeLa cervical cancer cells were downregulated when PROK2 expression is silenced by shPROK2.

Upregulation of matrix metalloproteinases (MMPs) contributes to ECM remodeling progression, tumor cell invasion and metastasis. Malignant cells use MMPs to change ECM structure, migrate and invade surrounding tissues and the circulatory system to reach the metastasis sites [17]. MMP15 is showed be associated with the high degree of malignancy, aggressiveness and survival prognosis. MMP15 is high expressed in patients with acute myeloid leukemia (AML). MMP15 expression is correlated with the clinicopathologic characteristics of AML patients. High MMP15 expression is significantly correlated with a short OS for all AML patients [20]. MMP15 positively correlates to the angiogenesis of human esophageal cancer. The OS of patients with low MMP15 protein expression is better that those with MMP15 protein expression [21]. MMP15 is reported to be associated with prostate cancer progression, using the expression analysis of human prostatic tissues [22]. In the present study, we first show that MMP15 is closely regulated by PROK2. MMP15 is significantly overexpressed in the tumor tissue. Cervical cancer patients with high MMP15 expression have a shorter OS than those with low MMP15 expression.

Several studies suggest that MMP15 acts as a critically intermediate regulator, and then contributes to tumor progression. MMP15 mediates HBXIP-facilitated migration, invasion and metastasis of hepatocellular carcinoma cells [7]. MMP15 mediates LncRNA MAFG-AS1-promoted the aggressiveness of breast carcinoma [23], and the migration and invasion of non-small-cell carcinoma (NSCLC) [24]. Upregulation of MMP15 by HLA-G might result in invasiveness or metastasis of ovarian cancer [25]. In the present study, we demonstrated that MMP15 is closely regulated by PROK2 in the expression of mRNA and protein. Cervical cancer cells transfected with PROK2 shRNA had a significant decrease in the transcription and translation of MMP15. PROK2-expressing vector reversed PROK2 shRNA-inhibited MMP15 expression, migration and invasion in human cervical cells. By Spearman correlation coefficient analysis, MMP15 was positively correlated with PROK2 expression. The present study suggests that PROK2 acts a critical role in the progression of human cervical cancer. PROK2 was expressed in higher amounts in human cervical cancer tissues compared with adjacent normal tissues. PROK2 was correlated with low OS and DFS in patients with cervical cancer.

A previous study demonstrated that PKRA7 is a PROK2 antagonist, which blocking the PROK2 expression, and consequently suppressing the tumorigenic and angiogenesis abilities of PROK2 and its downstream significantly pathways in glioma and pancreatic cells [10]. In another study, PKRA7 was administered intraperitoneally into collagen-induced arthritis (CIA) mice, and significantly inhibited the severity of arthritis [26]. Based on these evidences, PKRA7 as a candidate target reagent for cancer therapy. In future research will be focus on the antitumor effect and novel pathways of PKRA7 (PROK2 antagonist) in human cervical cancer, and it may be important implications for the development of molecular target drug against human cervical cancer.

## 4. Materials and Methods

### 4.1. Chemical and Reagent

CCK8 kit was purchased from Sigma-Aldrich (St. Louis, MO, USA). The primary antibodies against β-actin (sc-69879, 1:5000) was purchased from Santa Cruz Biotechnology (Santa Cruz, Dallas, TX, USA). Antibodies against PROK2 (24906-1-AP, 1:1000) was purchased from BioTools Co. Ltd. (Taipei, Taiwan). Antibodies against MMP15 (130522, 1:500) was obtained from Novus Biologicals (Briarwood, CO, USA) and horseradish peroxidase (HRP)-conjugated anti-mouse (AP124P, 1:10,000) were purchased from Merck Millipore (Burlington, MA, USA).

### 4.2. Human Cervical Tissue Array and TCGA Database Analysis

A human cervical tissue array (CR805) containing 70 cases of advanced stage human cervical cancer patients and 10 normal tissue samples was purchased from US Biomax Inc. (Rockville, MD, USA). The GEPIA dataset (http://gepia.cancer-pku.cn/index.html) from the TCGA database contains clinical data of 306 cervical tumor tissues and 13 normal tissues used to analyse the PROK2 mRNA expression. The PROK2 were detected with advanced stage (stage I~IV). Using the Kaplan-Meier analysis (https://kmplot.com/analysis/), the 304 patients were divided the low and high expression group by measuring the median of PROK2 and MMP15 mRNA expression as a cutoff. Spearman’s correlation analysis to determine the correlation between PROK2 and MMP15 expression.

### 4.3. Cell Lines

The human cervical cancer cell line HeLa and C33A cells were purchased from the Bioresources Collection and Research Center (Hsinchu, Taiwan). SiHa cells were kindly provide from Dr. Jiunn-Liang Ko Laboratory (Institute of Medicine, Chung Shan Medical University). HeLa cells cultured in DMEM/F12 medium (Invitrogen, Carlsbad, CA, USA), C33A and SiHa were culture in Dulbecco’s modified Eagle’s medium (DMEM) medium (Invitrogen), and supplemented with 10% fetal bovine serum (FBS; Gibco/Invitrogen, Waltham, MA USA) and 1% penicillin/streptomycin (HyClone, Logan, UT, USA) incubated at 37 °C in 5% CO_2_ humidified atmosphere.

### 4.4. shRNA for Knockdown Endogenous PROK2 and Transfection Assay with PROK2 Plasmid

For PROK2 knockdown, PROK2 small hairpin RNA plasmid (PROK2-shRNA-pLKO.1) was purchased from RNAi core of Academia Sinica (Taipei, Taiwan). The PROK2 targeting sequences are, 5′-CCG GTC AAC TTT CCA AGT AAC ATT TCT CGA GAA ATG TTA CTT GGA AAG TTG ATT TTT TG-3′, The pLKO.1-Luc as scrambled control. The lentivirus particles were produced in HEK-293T cells transfected with pMDG, pCMVΔR8.91 and pLKO-shRNA-containing plasmids. HeLa cells were infected with the lentivirus and stable cells (The HeLa-shPROK2 cells) were established by puromycin selection (2 μg/mL). The HeLa-shPROK2 cells transfected with pCMV scrambled (3 μg) plasmid or pCMV-PROK2 plasmid (3 μg) in Opti-MEM medium (Thermo Fisher Scientific, Waltham, MA, USA) and added to 9 μL of jetPEI^®^ transfection reagent (Polyplus^®^ Transfection, New York, NY, USA). Transfection for 48 h, the cells were lysed with extraction buffer for further cell experiments.

### 4.5. Cell Proliferation Assay

The HeLa-Luc or HeLa-shPROK2 cells were counted 8 × 10^3^/100 μL and seeded in 96 well plates (Greiner Bio-one, Frickenhausen, Germany). After incubation for 24 and 48 h, cells were cultured with fresh medium containing the CCK8 solution (100 μL/well) for 2 h. The cell viability was detected at 405 nm by using a Multiskan MS ELISA reader (Labsystems, Helsinki, Finland). 

### 4.6. Cell Cycle Distribution by Flow Cytometry

Cell cycle assay as previously report [27]. HeLa-Luc or HeLa-shPROK2 cells were collected and fixed with 75% ice ethanol for overnight. Then, these fixed cells were stained with PI reagent for 20 min. Cell DNA content was measured by Muse Cell Analyzer flow cytometry and outcome data further was analyzed by the Muse^®^ Cell Analyzer Assays (Millipore, Darmstadt, Germany).

### 4.7. Migration and Invasion Assay

The capability of *c*ellular migration and invasion was measured as described previously [28]. Briefly, cells seeded onto the filter inserts (8-μm pores) pre-coated with or without Matrigel for the invasion assay and migration assay, respectively. The cells migrating or invading to the lower side of the filter insert were stained with 5% Giemsa reagent and counted at 200× magnification. Four microscopic fields were counted for each filter, and each sample was assayed in triplicate.

### 4.8. Quantitative Reverse-Transcription Polymerase Chain Reaction (qRT-PCR)

Total RNA was isolated from cultured cells was performed as described previously [29]. The qRT-PCR primer was shown as PROK2: forward 5′-TTC ACA CCC AAC TTT AAT CCA CC-3′ and reverse 5′-TCC ATA GGG AGG TCA TAA TCA CC-3′, MMP-15: forward 5′- AGG TCC ATG CCG AGA ACT G-3′ and reverse 5′- GTC TCT TCG TCG AGC ACA CC-3′, GAPDH: forward 5′-CAT CAT CCC TGC CTC TAC TG-3′ and reverse 5′-GCC TGC TTC ACC ACC TTC-3′. The relative mRNA expression of PROK2 or MMP15 were calculated using 2^−∆∆Ct^ method.

### 4.9. Western Blotting Analysis

Western blotting analysis was performed as described previously [30]. Briefly, total proteins were harvested from these cells lysed with lysis buffer with protease/phosphatase inhibitor (Roche, South San Francisco, CA, USA). Following the lysate were sonicated on ice and the supernatant was collected by centrifugation at 12,000× *g* for 15 min at 4 °C. Equal amounts of total protein (20 µg) were subjected to 10~12% SDS-PAGE for protein separation, and then transferred onto PVDF membrane (Life Technologies, Carlsbad, CA, USA). The blocked membranes were incubated with target primary antibodies and followed by the incubation of secondary antibodies for detecting antibody-bound protein bands using the Luminescent Image Analyzer LAS-4000 mini.

### 4.10. Statistical Analysis

Statistical analysis was performed in GraphPad Prism v.5 (GraphPad Software, Inc., San Diego, CA, USA). The unpaired 2-tailed Student’s t-test and analysis of variance (ANOVA) was analysed to compare the significance of differences. * *p* < 0.05,** *p* < 0.01. Kaplan–Meier survival analysis was analyse the significance of low or high expression of PROK2/MMP15 in overall survival rate (OS) and (E) disease free survival rate (DFS) of human cervical patients. The correlations of PROK2/MMP15 were measured using Spearman’s correlation analysis.

## 5. Conclusions

PROK2 knockdown in cervical cancer cells considerably suppressed the capacity in cellular migration, invasion, and MMP15 expression. Inhibition of PROK2 might improve the treatment effect of cervical cancer. PROK2 might acts as a potential predictor and molecular target for cervical cancer therapy.
